# Nairobi sheep disease virus: an emerging threat with unresolved pathogenesis and zoonotic potential

**DOI:** 10.1128/jvi.00330-26

**Published:** 2026-04-29

**Authors:** Xue-Lian Zhang, Meng-Hang Wang, Jia-Hao Hu, De-Xin Liang, Xin Yin, Jian-Wei Shao

**Affiliations:** 1 School of Animal Science and Technology, Foshan University, Foshan, China; 2 State Key Laboratory of Animal Disease Control and Prevention, Harbin Veterinary Research Institute of Chinese Academy of Agricultural Sciences 687216 , Harbin, China; Indiana University Bloomington, Bloomington, Indiana, USA

**Keywords:** Nairobi sheep disease virus, molecular pathogenesis, geographical expansion, transmission dynamics, One Health, control strategies

## Abstract

Nairobi sheep disease virus (NSDV), a tick-borne orthonairovirus, causes severe hemorrhagic disease in small ruminants with mortality rates reaching 90% in naïve populations. Its recent emergence in China, the world’s largest small ruminant producer, marks a significant eastward expansion and poses immediate threats to global food security and animal health. This escalating crisis, however, stands in stark contrast to critical gaps in understanding the virus’s molecular pathogenesis. The mechanisms governing cellular entry, innate immune evasion, and induction of lethal vascular pathology remain poorly characterized, severely hampering countermeasure development. This review synthesizes the current epidemiological landscape of NSDV and delineates key knowledge gaps in its molecular biology. We place particular emphasis on the unexplored viral life cycle and the host response and conclude by proposing a prioritized research agenda aimed at bridging these mechanistic gaps. Within a One Health framework, such efforts are essential to mitigate the veterinary, economic, and potential zoonotic impacts of this emerging arthropod-borne threat.

## INTRODUCTION

Nairobi sheep disease virus (NSDV), a member of the genus *Orthonairovirus* within the family *Nairoviridae*, is the etiological agent of Nairobi sheep disease (NSD), an acute, hemorrhagic syndrome in sheep and goats (1). First identified in Kenya in 1910, this tick-borne pathogen is characterized by high fever, hemorrhagic gastroenteritis, and abortion, with mortality rates reaching 90% in susceptible populations (2). Historically endemic to East Africa and South Asia, NSDV imposes substantial economic burdens on small ruminant production in these regions (2–8), warranting its classification as a notifiable disease by the World Organisation for Animal Health (WOAH) (9).

The established geographical range of NSDV has been greatly expanded by its recent emergence in China. Conclusive evidence from virus isolation and serological surveillance confirms a significant eastward expansion of the virus (10). This breach into the world’s largest small ruminant producer poses substantial threats to global food security and animal health. Compounding this veterinary emergency is the demonstrated zoonotic potential of NSDV, which endangers abattoir workers, veterinarians, and rural populations (4, 7, 11–14), raising biosafety concerns comparable to those associated with its notorious relative, Crimean-Congo hemorrhagic fever virus (CCHFV).

This evolving threat landscape contrasts starkly with persistent deficits in understanding the virus’s fundamental biology. Despite over a century of recognition, the molecular mechanisms governing NSDV infection, from cellular entry and replication to the induction of lethal vascular pathology, remain largely uncharacterized. This knowledge gap critically impedes the development of robust diagnostics, effective vaccines, and targeted antiviral therapies. This review aims to synthesize the current epidemiological status of NSDV, critically examine nascent efforts to decipher its molecular pathogenesis, and outline a definitive research agenda to address these gaps and mitigate the impact of this emerging arthropod-borne threat.

## AN EXPANDING GEOGRAPHY AND A PERSISTENT MOLECULAR VOID

**Fig 1:**
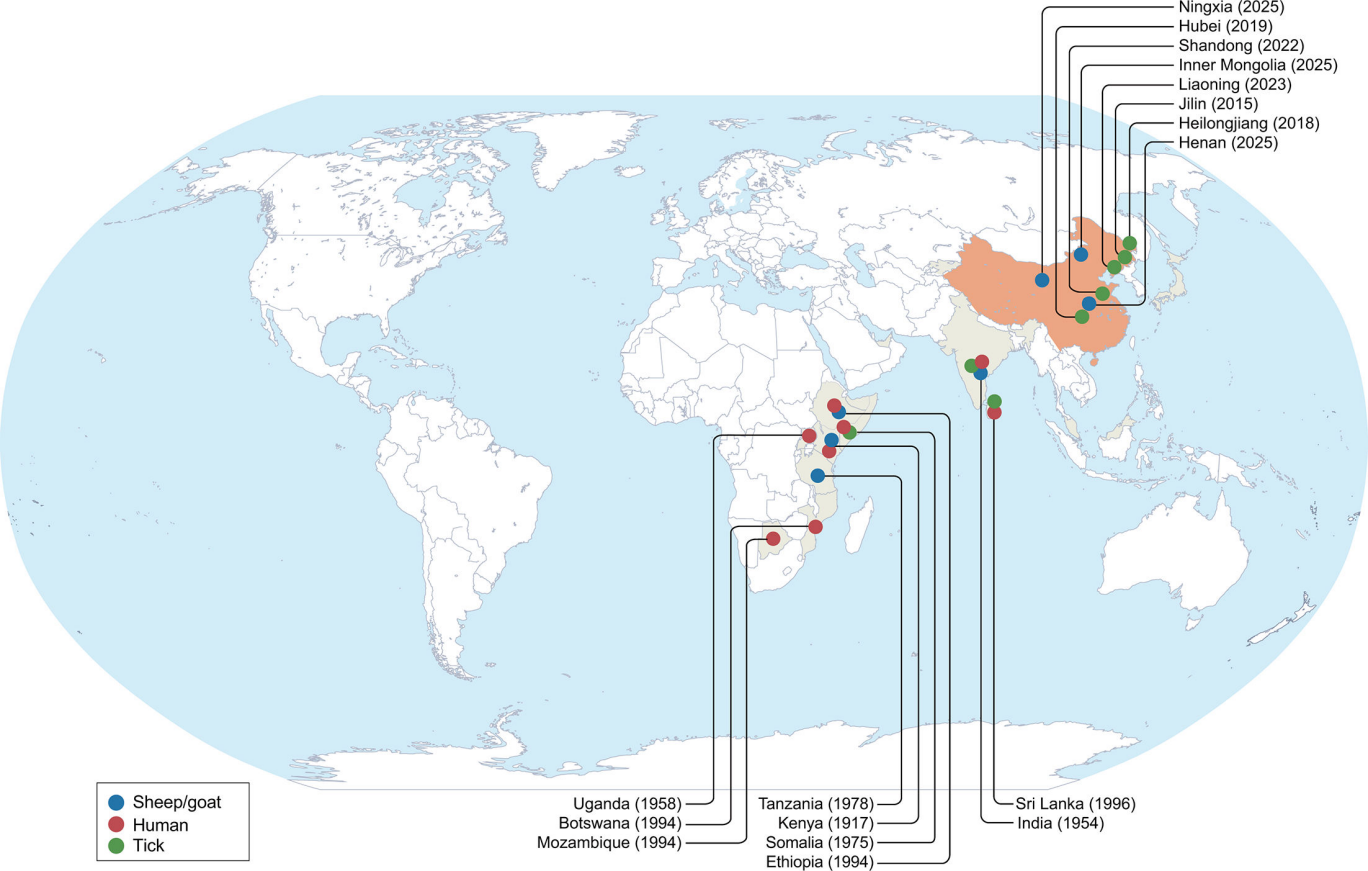
Historical and current geographical distribution of NSDV based on documented evidence. The map depicts the historically recognized endemic range (light gray) and newly established foci in China (orange). Years in parentheses indicate the year of first documented detection in each location. Symbol shapes denote the host source: blue circles, sheep/goat; red squares, human; green circles, tick. The geographical distribution illustrates a significant eastward expansion from the virus’s historically recognized range in East Africa and South Asia into East Asia, with multiple provinces in China now showing evidence of NSDV circulation. The clustering of detections across a broad latitudinal gradient in China suggests widespread establishment facilitated by the invasive tick vector *Haemaphysalis longicornis*. The base map was created with Surfer (v4, Golden Software) and annotated with Adobe Illustrator CC 2017.

NSD was first observed in Nairobi, Kenya, in 1910, and its causative agent, NSDV, was first isolated in 1917 (3). Historically, NSDV was endemic to East African countries, including Uganda, Ethiopia, Somalia, Tanzania, and Rwanda (2, 5–8). More recently, Ganjam virus, the Asian variant of NSDV isolated in India in 1954 and later documented in Sri Lanka, has been recognized as a variant of NSDV (4, 15, 16). The confirmed establishment of NSDV in China represents a pivotal development in its epidemiology (Fig. 1). This emergence is supported by accumulating evidence, ranging from initial RNA detection in *Haemaphysalis longicornis* ticks to the isolation of the MZ-18 strain from naturally infected small ruminants in Henan province, which subsequently demonstrated severe pathogenicity in experimental infections (10, 17–23). A serological survey revealing a 4.1% RNA positive rate and a 29.9% seropositivity rate further confirms substantial, frequently subclinical, and viral circulation (10). This geographical breach is facilitated by converging factors, including dense populations of susceptible small ruminants in China’s predominant small-scale farming systems (24) and the ubiquitous distribution of the highly adaptable and invasive tick vector *H. longicornis* across the country (25). Genetic evidence solidifies this picture, with phylogenetic analyses showing intermingled Chinese NSDV sequences from ticks and livestock forming distinct subclades, indicating multiple sustained transmission cycles (10).

Despite successful geographical expansion, understanding of NSDV’s molecular life cycle remains critically limited. The viral components responsible for infection and pathogenesis are largely uncharacterized: receptors mediating broad cellular tropism remain unidentified; functions of putative virulence factors like the ovarian-tumor (OTU) domain have been demonstrated initially but need further experimental validation (26–29). The mechanistic drivers of lethal hemorrhagic pathology, including the dysregulated cytokine storm that drives endothelial dysfunction, vascular leakage, and coagulopathy, remain unknown. This disparity between an expanding ecological presence and stagnant molecular understanding defines the current crisis in NSDV research. Current dependence on acaricide-based tick management is increasingly challenged by resistance, highlighting that bridging this molecular void is not merely an academic exercise but an essential step in mitigating this emerging threat.

## DECONSTRUCTING THE EXPANSION: ECOLOGICAL MECHANISMS AND DRIVERS

The recent establishment of NSDV in China reflects a convergence of ecological factors that have facilitated its eastward expansion. Central to this process is the invasive tick species *H. longicornis*, whose remarkable adaptability enables it to function as both a competent vector and efficient reservoir across diverse Chinese ecosystems (25). Once predominantly confined to its native range in East Asia, including China, Japan, Korea, and eastern Russia, *H. longicornis* has demonstrated a significant capacity for invasion (25). It became well established in Australia, New Zealand, and several Pacific islands years ago and has recently emerged as a pest in multiple U.S. states (25, 30). Currently, this species is present in at least 10 countries, with its primary distribution spanning eastern Asia, the United States, Australia, and New Zealand (25). Within China, its range was historically confined to eastern coastal provinces and northeastern regions (31, 32). However, climate change has broadened its suitable habitats both latitudinally and longitudinally. Ecological niche modeling predicts further expansion into northwestern China, including Xinjiang and Inner Mongolia, as well as into central Asian countries such as Kazakhstan and Mongolia, and the highlands of Southeast Asia, including northern Vietnam, Laos, and Myanmar (25, 33). The seasonal activity window of *H. longicornis* in temperate China has historically been restricted to spring and early summer, from April to July, lasting approximately 90 days. Warmer temperatures have advanced the onset of tick activity by 10–15 days and extended the active season into September–October, broadening the transmission window by 3–4 weeks (34).

Concurrent transformations of agricultural landscape in China, particularly the intensification of small ruminant production and the expansion of smallholder farming systems, have created ideal conditions for viral establishment by providing dense populations of immunologically naïve hosts. This ecological susceptibility is compounded by the complex nature of viral invasion. Genetic evidence reveals sustained local circulation of NSDV within China, with viral strains forming distinct subclades that suggest ongoing transmission and adaptation within diverse ecological niches. Together, these forces, including a highly adaptable competent vector, climate-driven habitat expansion, agricultural intensification, and human-mediated dispersal, represent an ecological convergence that has effectively dismantled the geographical barriers once confining NSDV to its traditional ranges. This convergence has not only created unprecedented opportunities for NSDV range expansion but will also shape both the virus’s trajectory and our capacity to respond, through the lens of its molecular architecture and host interactions governing pathogenesis.

## GENOMIC BLUEPRINT AND EVOLUTIONARY CONSTRAINTS: A PLATFORM FOR PATHOGENESIS

**Fig 2:**
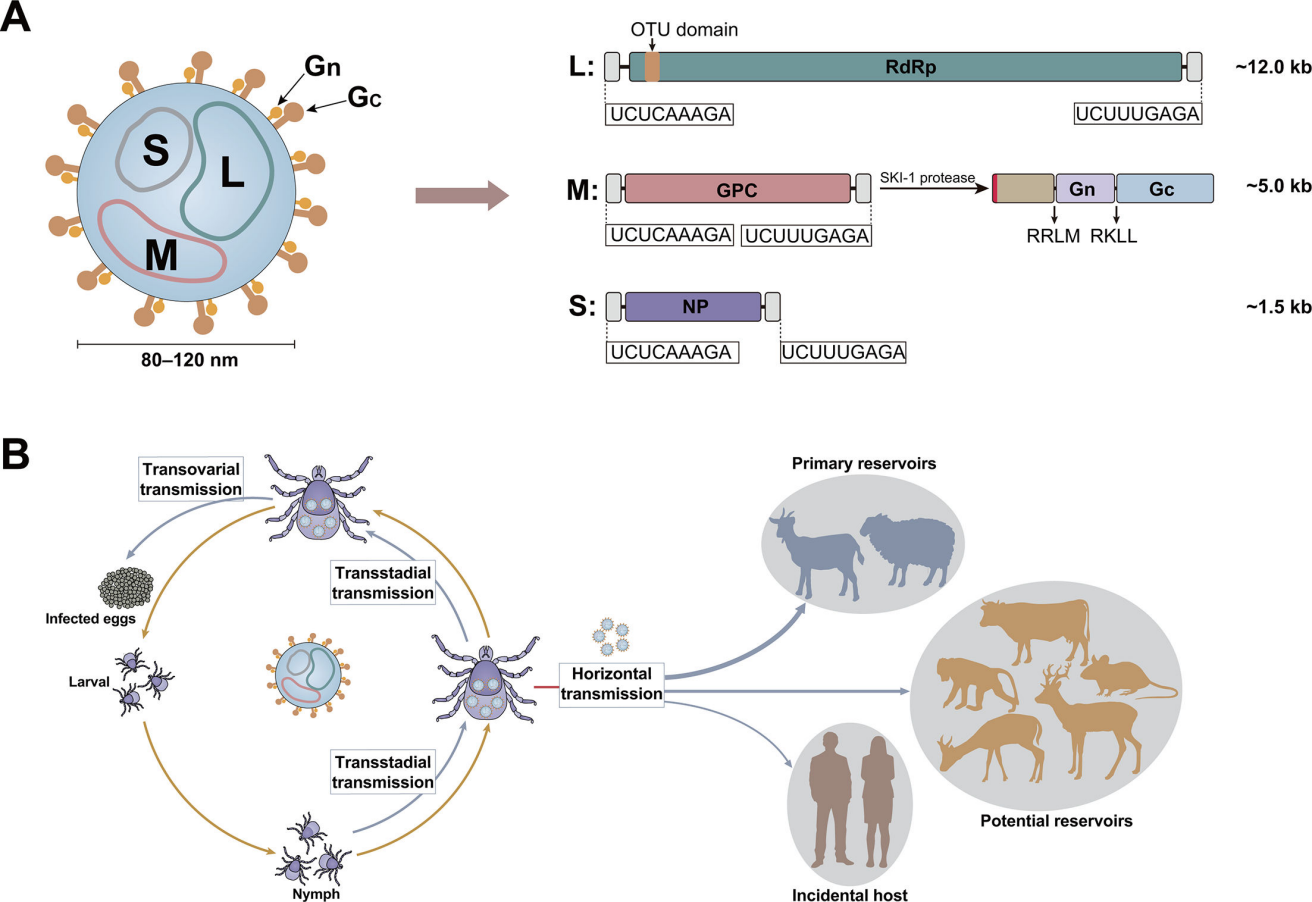
Schematic representation of NSDV structure and its enzootic transmission cycle. (**A**) Virion architecture and genomic organization. The virion model (left) depicts the envelope embedded with Gn/Gc glycoproteins and the internal ribonucleocapsid. The genomic structure (right) shows the three negative-sense RNA segments (L, M, and S) with their approximate lengths and encoded proteins: RNA-dependent RNA polymerase (RdRp), which contains an N-terminal ovarian-tumor (OTU) domain with deubiquitinase and deISGylase activity, the glycoprotein precursor (GPC), cleaved into Gn and Gc by the host SKI-1 protease, and nucleoprotein (NP). (**B**) The maintained transmission cycle of NSDV among ticks and vertebrate hosts. The virus is sustained through transovarial and transstadial transmission in ixodid ticks and an enzootic tick–vertebrate–tick cycle. Arrow thickness represents transmission efficiency and viral load: the thickest arrows indicate efficient transmission to and from domestic small ruminants (sheep/goats); medium arrows indicate wild animals (e.g., blue duiker) as potential reservoirs; thin arrows indicate incidental human infections.

NSDV possesses a tripartite, negative-sense RNA genome characteristic of nairoviruses (1). The large (L) segment encodes the RNA-dependent RNA polymerase (RdRp); the medium (M) segment encodes a glycoprotein precursor that is cleaved to form the structural glycoproteins Gn and Gc; and the small (S) segment encodes the nucleoprotein (N) (Fig. 2A). All genomic segments terminate in highly conserved pan-handle sequences (3′-UCUCAAAGA…UCUUUGAGA-5′), which facilitate the formation of a panhandle structure through terminal base pairing, enabling polymerase recognition and replication, a feature shared among orthonairoviruses (Fig. 2A).

**Fig 3:**
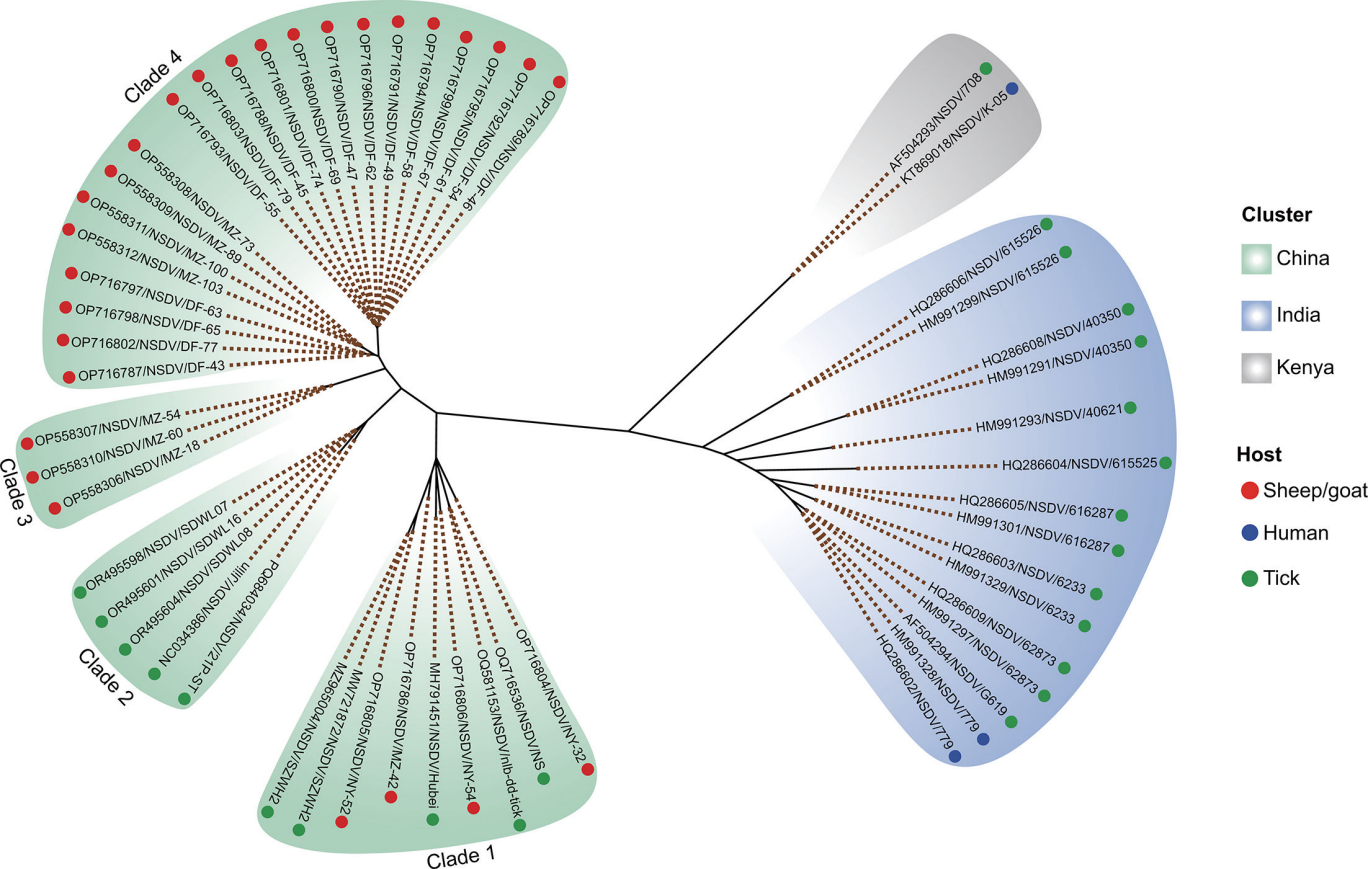
Phylogenetic relationships of NSDV strains based on complete S segment sequences. The maximum-likelihood tree illustrates the segregation of NSDV into distinct geographical clades (Kenya, India, and China). The clustering of Chinese sequences from both tick and livestock hosts into multiple, closely related subclades provides genetic evidence for sustained, independent transmission cycles within the region. Tip symbols indicate the host source.

Phylogenetically, NSDV strains segregate into distinct geographical clades corresponding to Kenya, India, and China, with Chinese sequences forming multiple subclades that indicate sustained local transmission (Fig. 3). The Chinese clade exhibits greater genetic diversity compared to the Kenyan and Indian clades. This diversity likely reflects sustained local circulation of NSDV within China, potentially shaped by adaptation to the diverse ecological niches encountered across the country’s heterogeneous landscape, including variations in host species and populations, climatic conditions, habitat types, and potentially distinct genetic subpopulations of the vector *H. longicornis*.

**Fig 4:**
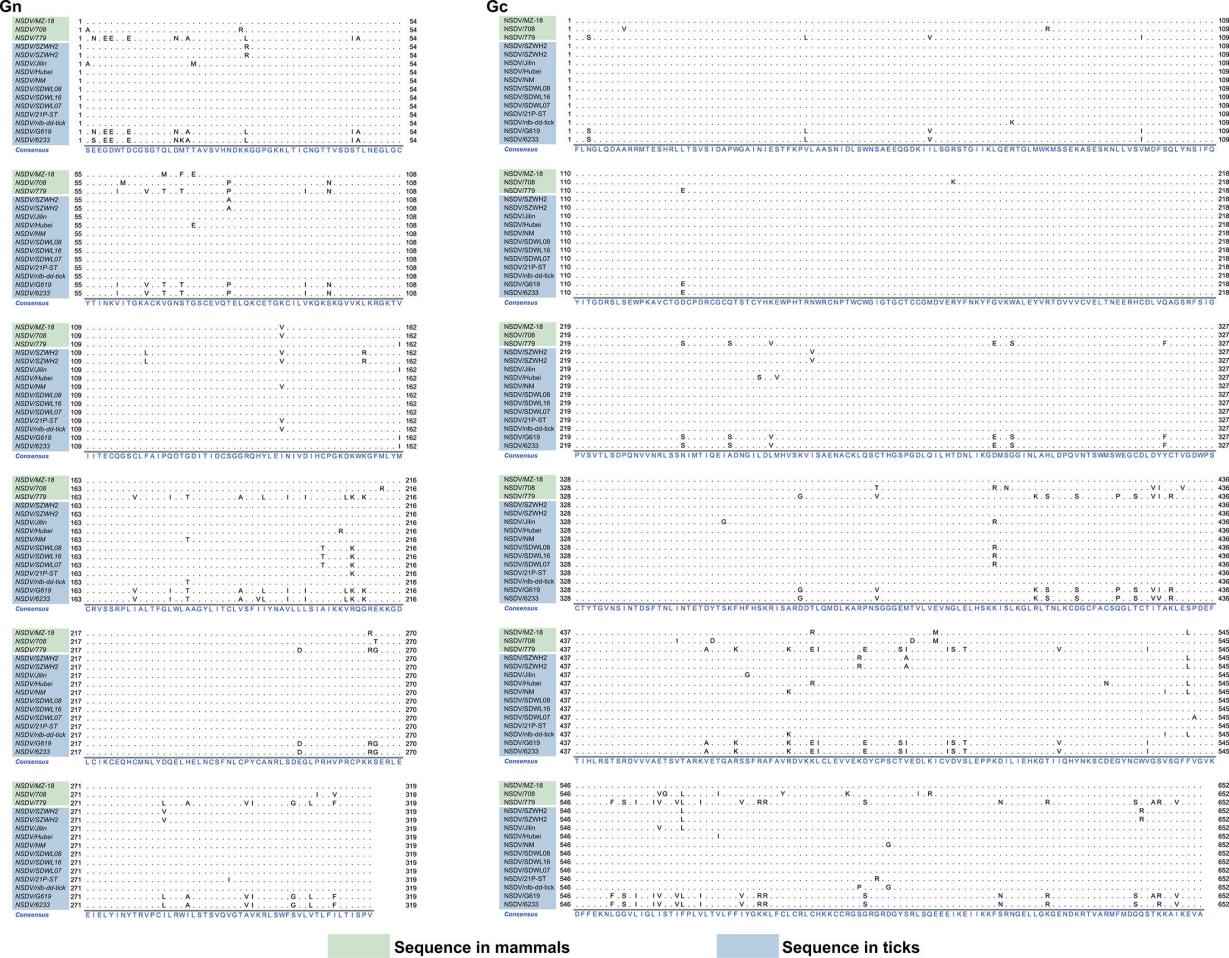
High sequence conservation of NSDV glycoproteins across global isolates. The multiple sequence alignment of deduced Gn and Gc glycoprotein sequences reveals a high degree of amino acid conservation (≥86% identity). This strong evolutionary constraint underscores the functional importance of these proteins in viral entry and membrane fusion, positioning them as prime targets for broad-spectrum vaccine and therapeutic development. The alignment was generated using the ClustalW algorithm in MEGA XI and visualized with Jalview (v2.11.1.4).

Comparative genomics reveals striking amino acid conservation, with 86% or greater identity in Gn and Gc glycoproteins across strains from ticks and mammalian hosts (Fig. 4), suggesting strong functional constraints on these proteins, which are critical for host cell attachment and entry (1). This genomic stability provides the essential toolkit that enables the virus to initiate infection and maintain efficient transmission cycles. It also offers promising targets for broad-spectrum vaccine development and monoclonal antibody therapies, as interventions targeting these conserved epitopes would likely be effective against diverse circulating strains.

## THE TICK–LIVESTOCK–HUMAN INTERFACE: A TRIAD OF UNANSWERED QUESTIONS

The genetic repertoire of NSDV is deployed within a complex ecological context (Fig. 2B). The virus is maintained through an enzootic tick–vertebrate–tick cycle (5), with *H. longicornis* serving as the primary vector and reservoir in China through efficient transstadial and transovarial transmission (17–22, 35). The primary vector varies geographically: in East Africa, *Rhipicephalus appendiculatus* is the principal vector; in South Asia, *Haemaphysalis intermedia* plays this role, though NSDV has also been isolated from *Haemaphysalis wellingtoni* and *Haemaphysalis turturis* (2).

The virus exhibits a paradoxical host range. Under natural conditions, clinical disease is almost exclusively observed in sheep and goats, likely reflecting the feeding preferences of the primary tick vectors: *H. longicornis* in China and *R. appendiculatus* in East Africa predominantly feed on small ruminants, limiting opportunities for spillover to other species. A few fatal cases have been reported among blue duikers (*Cephalophus monticola*) in zoological or wild settings (36). However, this African forest antelope species is not native to China and is therefore unlikely to serve as a natural reservoir in the newly emerged Chinese foci. In contrast, the potential role of local wildlife in NSDV maintenance in China remains unexplored. These observations suggest a relatively narrow vertebrate host range under natural conditions.

However, this contrasts sharply with its demonstrated zoonotic potential and its ability to infect diverse cell types *in vitro*. In experimental settings, NSDV has been shown to infect cattle (37), African field rat (*Arvicathus abysinicus nubilans*), and langur monkeys (*Presbytis entellus*) (36), and laboratory rodents such as hamsters and mice (9), as well as various cell lines, including human (A549 and Huh7), primate (Vero), and rodent (BHK-21) cells (10). It should be noted that experimental infections often involve higher viral inocula and parenteral routes that bypass natural transmission barriers, which may account for the expanded host range observed under laboratory conditions. Furthermore, caution is warranted when extrapolating from *in vitro* cell line data to *in vivo* host range predictions, as cell lines such as A549, Vero, and BHK-21 are broadly permissive to many viruses due to immortalization-induced alterations or deficiencies in innate immune pathways. Taken together, these findings indicate that the molecular machinery required for viral entry and replication is broadly conserved across species, whereas clinical outcomes are distinctly species dependent.

Human infections with NSDV, though rarely reported, provide critical insights into its zoonotic potential. Documented cases include laboratory-acquired infections resulting from needlestick or aerosol exposure, which presented with acute febrile illness, with temperatures reaching 39°C–40°C, along with severe headache, myalgia, and nausea, all resolving within 5–7 days (36). In addition, serological evidence of natural tick-borne infections has been documented in occupationally exposed individuals in India, Kenya, and Sri Lanka (4, 7, 11–14). Laboratory-acquired infections may be more severe, possibly due to a higher inoculum or parenteral route of exposure, but no fatalities have been reported. Despite efficient replication of NSDV in human cell lines *in vitro*, human disease appears uniformly mild compared to the devastating outcomes in sheep, suggesting host-specific differences in pathogenesis (36). The molecular basis for this cross-species potential, likely involving interactions between conserved viral glycoproteins and ubiquitous host factors, remains a critical unanswered question demanding investigation within a One Health framework.

## FROM FEVER TO HEMORRHAGE: THE UNRESOLVED CLINICAL TRAJECTORY

Successful transmission of NSDV culminates in devastating clinical outcomes. In susceptible small ruminants, NSD manifests as an acute febrile illness that rapidly progresses to severe hemorrhagic gastroenteritis, often proving fatal within 1 week (9). Mortality rates range from 30% to 90%, depending on host factors such as breed and immune status, as well as viral strain virulence. Imported breeds experience higher mortality, up to 90%, compared to indigenous breeds, which have mortality rates of 30%–50% (9, 36). Characteristic postmortem findings include hemorrhagic zebra striping in the colon, though these lesions are not pathognomonic and may be absent in peracute cases (9, 36). The systemic hemorrhagic diathesis and multi-organ failure closely mirror the vascular pathology observed in human CCHFV infections, suggesting shared but poorly understood mechanisms of endothelial dysfunction and dysregulated immune activation. This clinical picture underscores the necessity of advancing beyond descriptive pathology to molecular inquiry into disease drivers.

## UNLOCKING THE BLACK BOX: VIRAL ENTRY, REPLICATION, AND THE STORM WITHIN

The clinical manifestations of NSD originate in molecular interactions between the virus and its host. Viral entry begins with engagement of the conserved Gn and Gc glycoproteins with host cells, though the specific cellular receptors conferring broad tropism remain unidentified. This knowledge gap contrasts with progress in related viruses, particularly the identification of low-density lipoprotein receptor (LDLR) family members as entry factors for CCHFV and other arboviruses (38–44). The recurrent exploitation of this receptor family, especially by the closest relative of NSDV, positions LDLR-related receptors as priority candidates for NSDV entry. Identifying these receptors is a critical bottleneck, and their resolution is essential for developing entry inhibitors. The LDLR family, as host factors essential for entry, represents a potential target for host-directed antiviral strategies, such as soluble receptor decoys or compounds that disrupt virus–receptor interaction.

Following entry, the virus initiates replication, during which a pivotal molecular virulence determinant, the OTU domain of the viral L protein, comes into play. This domain in NSDV functions as a potent deubiquitinase and deISGylase. This activity was initially predicted based on robust functional studies of its close ortholog in other orthonairoviruses, especially CCHFV (45–48), and was subsequently confirmed by experimental evidence demonstrating that NSDV exhibits significant deubiquitinating and deISGylating activity (26–29). Its isolated OTU domain actively inhibits both the induction of and cellular response to interferons (49). This enzymatic activity represents a key molecular weapon in the virus–host arms race, postulated to disarm the host’s critical innate interferon response by stripping activating ubiquitin and ISG15 modifications from key signaling proteins like RIG-I and STAT1 (45, 48, 50, 51). Beyond this direct antagonism of innate immunity, recent transcriptomic profiling has revealed that NSDV further subverts host cell biology by exploiting autophagy as a proviral mechanism to enhance viral replication (52). Moreover, SKI-1/S1P protease has been identified as a critical host factor for NSDV infectivity (53). Nevertheless, experimental validation of this function in NSDV and the identification of its specific host targets using more sophisticated approaches, such as reverse genetics, remain top research priorities.

These processes culminate in lethal vascular pathology, a storm within the vessel where immunopathological events converge to drive endothelial dysfunction, vascular leakage, and disseminated intravascular coagulation. NSDV infection triggers a dysregulated cytokine storm: infected monocytes and macrophages release pro-inflammatory cytokines such as TNF-α, IL-6, and IL-10, which in turn drive endothelial activation, increased vascular permeability, a pro-coagulant shift, and disseminated intravascular coagulation (54–58). This immunopathogenic profile closely mirrors that of severe CCHFV infection (55, 59, 60), suggesting shared vascular disruption strategies among related nairoviruses. The relative contributions of direct viral cytopathy versus immunopathology to clinical outcomes remain a central unresolved question. Answering this question has direct implications for developing targeted supportive therapies, such as TNF-α inhibitors or JAK/STAT pathway modulators, to mitigate vascular damage (55, 60).

## BRIDGING THE GAP: FROM MECHANISTIC INSIGHTS TO NEXT-GENERATION COUNTERMEASURES

Several factors have historically constrained NSDV research. The virus has been disproportionately neglected as a veterinary pathogen affecting low- and middle-income countries. Its BSL-3 containment requirements limit laboratory access. There has also been a reliance on costly, logistically challenging natural host experiments due to the lack of standardized small animal models. However, modern tools now offer unprecedented opportunities to overcome these limitations. Reverse genetics systems will enable targeted mutagenesis of suspected virulence determinants. CRISPR screens can systematically identify host dependency factors. Advanced organoid models can recapitulate key aspects of vascular pathology without extensive animal experimentation. These approaches, combined with growing awareness of NSDV’s global threat, position the field for accelerated discovery.

Addressing the growing NSDV threat requires a fundamental shift from descriptive studies to mechanistic inquiry. A cohesive, prioritized research agenda should be built upon a foundation of identifying the critical host factors enabling infection, using unbiased functional genomics and proteomics approaches (Table 1). This includes genome-wide CRISPR knockout screens in permissive cell lines to identify viral receptors and host dependency factors; proximity labeling such as BioID or APEX coupled with mass spectrometry to map virus–host protein interaction networks; and single-cell multi-omics, including scRNA-seq and scATAC-seq, to dissect cellular heterogeneity in the response to infection (38, 61, 62).

**TABLE 1:** Prioritized research agenda for NSDV with key experimental strategies and expected outcomes

Priority tier	Research objective	Key experimental strategies	Expected outcomes
Tier 1 (highest priority)	1.1 Receptor identification	(i) Genome-wide CRISPR knockout screens (ii) Gn/Gc pull-down MS (iii) Candidate receptor validation	(i) Identify functional receptors (ii) Enable entry inhibitor development
	1.2 OTU domain validation	(i) *In vitro* DUB/deISGylase assays (ii) Reverse genetics mutagenesis	(i) Confirm OTU function (ii) Identify critical residues
	1.3 Reverse genetics system	(i) Full-length cDNA clones (ii) Virus rescue and characterization	Enable gene function studies and vaccine development
Tier 2 (high impact)	2.1 Endothelial dysfunction	(i) Endothelial-immune cell co-culture (ii) Vascular organ-on-chip	(i) Establish vascular pathology models (ii) Reveal injury mechanisms
	2.2 Host response profiling	(i) Single-cell RNA sequencing (ii) Multiplex cytokine analysis	(i) Map host responses (ii) Identify pathogenic pathways
	2.3 Vector–virus interaction	(i) Tick cell lines and infection model establishment (ii) Tick salivary gland proteomics (iii) RNAi screens	(i) Define vector competence factors (ii) Transmission-blocking targets
Tier 3 (translational)	3.1 Vaccine platform	(i) Subunit vaccines (ii) Live-attenuated vaccines (iii) Viral vector vaccines	Obtain protective vaccine candidates
	3.2 Antiviral screening	(i) RdRp inhibitors, (ii) OTU protease inhibitors, (iii) Host-directed therapies	Identify lead compounds with antiviral activity
	3.3 Rapid diagnostics	(i) Antigen detection strips (ii) LAMP assays (iii) CRISPR-RPA assays	Field-deployable tests for early outbreak detection

This foundation work must be coupled with systematic functional characterization of key viral proteins, including the immune-evasive OTU domain, the entry-mediating glycoproteins, and the nucleoprotein, using robust reverse genetics systems in biologically relevant models. Parallel development of advanced pathogenesis models, such as endothelial organoids, will help delineate molecular mechanisms driving vascular leakage and dysregulated host responses.

Ultimately, success will be measured by the translational application of these insights into next-generation countermeasures. Priority areas include the development of broad-spectrum vaccines targeting conserved viral epitopes and antiviral therapies directed against the RdRp or OTU domain. A deep molecular understanding of NSDV is no longer a luxury but a necessity for safeguarding animal health, economic stability, and global public health security against this evolving threat.
